# Supplementary material for: Towards Novel Potential Molecular Targets for Antidepressant and Antipsychotic Pharmacotherapies
Source: Int J Mol Sci. 2023 May 30;24(11):9482. doi: 10.3390/ijms24119482 (PMC10253480; doi:10.3390/ijms24119482)
Supplement: Supplementary file 1 [file ijms-24-09482-s001.zip › ijms-2376804-supplementary.pdf]

Table S1. Number of active compounds and accuracy of prediction (IAP) for antidepressant and related mechanisms of action.

**Number**	**IAP**	**Predictable Activity Type**
19174	0.897	Antidepressant
3101	0.989	5 Hydroxytryptamine 1 agonist
1701	0.991	5 Hydroxytryptamine 1A agonist
5764	0.984	5 Hydroxytryptamine 1A antagonist
135	0.989	5 Hydroxytryptamine 1B agonist
1516	0.984	5 Hydroxytryptamine 1B antagonist
1518	0.985	5 Hydroxytryptamine 1D antagonist
7461	0.968	5 Hydroxytryptamine 2 antagonist
5262	0.979	5 Hydroxytryptamine 2A antagonist
1154	0.993	5 Hydroxytryptamine 2C agonist
2825	0.977	5 Hydroxytryptamine 2C antagonist
2432	0.986	5 Hydroxytryptamine 3 antagonist
2548	0.988	5 Hydroxytryptamine 6 antagonist
1272	0.985	5 Hydroxytryptamine 7 antagonist
6367	0.984	5 Hydroxytryptamine agonist
18747	0.967	5 Hydroxytryptamine antagonist
23	0.909	5 Hydroxytryptamine release stimulant
7398	0.985	5 Hydroxytryptamine uptake inhibitor
16	0.886	5 Hydroxytryptamine uptake stimulant
244	0.997	AMPA receptor agonist
2988	0.987	Acetylcholine M2 receptor antagonist
1604	0.979	Acetylcholine nicotinic agonist
2865	0.978	Acetylcholine nicotinic antagonist
4749	0.986	Adenosine A1 receptor antagonist
6432	0.985	Adenosine A2 receptor antagonist
4912	0.986	Adenosine A2a receptor antagonist
8976	0.977	Adenosine receptor antagonist
4131	0.983	Adrenaline uptake inhibitor
1069	0.987	Alpha 1 adrenoreceptor agonist
2759	0.973	Alpha 2 adrenoreceptor antagonist
1935	0.983	Alpha adrenoreceptor agonist
7610	0.971	Alpha adrenoreceptor antagonist
921	0.984	Amidase inhibitor
822	0.985	Androgen agonist
482	0.985	Benzodiazepine agonist
69	0.99	Benzodiazepine inverse agonist
2664	0.999	Beta 3 adrenoreceptor agonist
449	0.978	Beta 3 adrenoreceptor antagonist
5416	0.985	Beta adrenoreceptor agonist
69	0.998	Bombesin 1 receptor antagonist
4	1.000	Carnitine O-acetyltransferase stimulant
2153	0.996	Cholecystokinin B antagonist
1600	0.995	Corticotropin releasing factor 1 receptor antagonist
681	0.998	Corticotropin releasing factor antagonist
8375	0.983	Dopamine D2 antagonist
538	0.994	Dopamine D3 agonist
2896	0.983	Dopamine agonist

185	0.999	Dopamine autoreceptor agonist
217	0.987	Dopamine beta hydroxylase inhibitor
3932	0.985	Dopamine uptake inhibitor
3593	0.987	GABA A receptor antagonist
446	0.989	GABA B receptor antagonist
1112	0.966	GABA receptor agonist
74	0.997	GABA uptake inhibitor
177	0.850	Galanin receptor 3 antagonist
211	0.839	Galanin receptor antagonist
1212	0.996	Glutamate (mGluR2) antagonist
312	0.993	Glutamate (mGluR3) antagonist
3014	0.990	Glutamate (mGluR5) antagonist
71	0.954	Glutamate release inhibitor
30	0.992	Inositol monophosphatase inhibitor
11	0.969	Lipocortins synthesis antagonist
2623	0.972	MAO A inhibitor
3993	0.977	MAO B inhibitor
5366	0.964	MAO inhibitor
593	0.994	Melatonin agonist
1290	0.995	Melatonin antagonist
929	0.987	NMDA 2B receptor antagonist
27	0.999	NMDA receptor glycine site B antagonist
731	0.997	NMDA receptor glycine site antagonist
4539	0.992	Neurokinin 1 antagonist
1751	0.990	Neurokinin 2 antagonist
1515	0.993	Neurokinin 3 antagonist
2712	0.971	Neuropeptide Y antagonist
9	0.857	Neurotransmitter antagonist
14	1.000	Nicotinamide phosphoribosyltransferase stimulant
434	0.983	Nicotinic alpha4beta2 receptor antagonist
907	0.995	Nociceptin (N/OFQ) receptor antagonist
3884	0.970	Opioid kappa receptor antagonist
989	0.943	P-glycoprotein 1 inhibitor
2887	0.983	Phosphodiesterase 4B inhibitor
7501	0.980	Phosphodiesterase IV inhibitor
19589	0.958	Phosphodiesterase inhibitor
2217	0.953	Phospholipase A2 inhibitor
14	0.977	Sigma 1 receptor agonist
26	0.963	Sigma receptor agonist
2242	0.991	Sigma receptor antagonist
11475	0.960	Sodium channel blocker
3146	0.992	Substance P antagonist
322	0.991	Tryptophan 5 hydroxylase inhibitor
525	0.997	Vasopressin 1B antagonist

IAP – Invariant Accuracy of Prediction calculated by leave-one-out cross-validation. It is equal AUC value.

Table S2. Number of active compounds and accuracy of prediction (IAP) for antischizophrenic effect and related mechanisms of action.

Number	IAP	Predictable Activity Type
48	0.910	Antischizophrenic
7461	0.968	5 Hydroxytryptamine 2 antagonist
5262	0.979	5 Hydroxytryptamine 2A antagonist
2825	0.977	5 Hydroxytryptamine 2C antagonist
2432	0.986	5 Hydroxytryptamine 3 antagonist
2548	0.988	5 Hydroxytryptamine 6 antagonist
1272	0.985	5 Hydroxytryptamine 7 antagonist
244	0.997	AMPA receptor agonist
2030	0.992	AMPA receptor antagonist
1835	0.992	Acetylcholine M1 receptor agonist
411	0.997	Acetylcholine M4 receptor agonist
2284	0.988	Acetylcholine muscarinic agonist
1604	0.979	Acetylcholine nicotinic agonist
4131	0.983	Adrenaline uptake inhibitor
1069	0.987	Alpha 1 adrenoreceptor agonist
912	0.966	Alpha 2c adrenoreceptor antagonist
7610	0.971	Alpha adrenoreceptor antagonist
4992	0.917	Antioxidant
5416	0.985	Beta adrenoreceptor agonist
1242	0.963	Beta amyloid protein antagonist
4816	0.972	Butyrylcholinesterase inhibitor
3868	0.984	Cannabinoid CB1 receptor antagonist
5721	0.986	Cannabinoid receptor agonist
455	0.995	Catechol O-methyltransferase inhibitor
2153	0.996	Cholecystokinin B antagonist
9662	0.959	Cholinergic antagonist
591	0.997	Dopamine D1 agonist
1613	0.981	Dopamine D1 antagonist
8375	0.983	Dopamine D2 antagonist
538	0.994	Dopamine D3 agonist
3789	0.984	Dopamine D3 antagonist
2387	0.986	Dopamine D4 antagonist
10756	0.980	Dopamine antagonist
185	0.999	Dopamine autoreceptor agonist
588	0.996	Estrogen receptor beta agonist
885	0.994	Glutamate (mGluR2) agonist
114	0.993	Glutamate (mGluR3) agonist
1999	0.996	Glycine transporter 1 inhibitor
5057	0.993	Histamine H3 receptor antagonist
1660	0.977	Inducible nitric-oxide synthase inhibitor
6006	0.977	NMDA receptor antagonist
389	0.960	Nav1.3 sodium channel blocker
6191	0.983	Nav1.7 sodium channel blocker
788	0.979	Nav1.8 sodium channel blocker
1515	0.993	Neurokinin 3 antagonist
6147	0.989	Neurokinin antagonist
252	0.985	Neurotensin receptor antagonist

975	0.988	Nicotinic alpha7 receptor agonist
3094	0.970	Nitric-oxide synthase inhibitor
21	0.901	Nitric-oxide synthase stimulant
5265	0.984	Opioid mu receptor antagonist
5287	0.992	Phosphodiesterase 10A inhibitor
604	0.993	Phosphodiesterase 9A inhibitor
845	0.967	Phosphodiesterase I inhibitor
964	0.988	Prolyl endopeptidase inhibitor
14	0.977	Sigma 1 receptor agonist
1058	0.997	Sigma 1 receptor antagonist
585	0.998	Sigma 2 receptor antagonist
12	1.000	Sigma 3 receptor antagonist
2242	0.991	Sigma receptor antagonist
2739	0.993	Sphingosine 1-phosphate receptor 1 agonist
628	0.996	Sphingosine 1-phosphate receptor 3 agonist
186	0.987	Sphingosine 1-phosphate receptor 4 agonist
297	0.986	Sphingosine 1-phosphate receptor 5 agonist
311	0.991	Sphingosine kinase 1 inhibitor
629	0.970	Trace amine-associated receptor 1 agonist
102	0.980	Vanilloid 2 antagonist
195	0.992	Vesicle monoamine transporter 2 inhibitor
1616	0.981	p38 MAP kinase inhibitor

IAP – Invariant Accuracy of Prediction calculated by leave-one-out cross-validation. It is equal AUC value.
